# Healthcare professionals’ encountering Experience of the Youths with Non-suicidal Self-injury in Acute Psychiatric Ward

**DOI:** 10.1192/j.eurpsy.2022.1575

**Published:** 2022-09-01

**Authors:** A.-N. Pan, E.C.-L. Lin

**Affiliations:** National Cheng Kung University, Taiwan, Department Of Nursing, Tainan City, Taiwan

**Keywords:** encountering experience, narrative inquiry, Healthcare professionals, Non-suicidal self-injury

## Abstract

**Introduction:**

Non-suicidal Self-Injury (NSSI) refers to causing damage on body tissue without attending to death. It is mostly presented among the youths and not approved by the society. Studies nowadays have explored the perspectives, feelings or experience of the youths or healthcare professionals. However, negative feelings and misunderstandings toward each other remain from both sides.

**Objectives:**

The aim was to explore the encountering experience of the youths with NSSI and the healthcare professionals during the same hospitalization in a psychiatric acute ward.

**Methods:**

Qualitative study was employed by using narrative approach. In-depth interview was conducted for the youths with NSSI and their primary nurse and resident from a medical center in southern Taiwan.

**Results:**

Narratives from the patients and healthcare professionals showed that the youths seemed to be comfortable as encountering with the healthcare professionals’ caring. In contrast, the healthcare professionals’ struggles had been hidden inside and remained uneasy and unsolved. Two extreme experiences have been reported by the youths with NSSI: felt satisfied and understood about being cared vs. felt numbness and not been understood. Four kinds of experience were identified as: struggling on caring them, feeling confused and helpless, keeping a safe distance, and having contradicted values.

**Conclusions:**

This study found that the healthcare professionals suffer from varied aspects when encountering the youths with NSSI, which they often hid inside without expressing. Future improvement such as care guideline or staff’s support system should be built to decrease the negative effects inside the healthcare professionals’ mind.

**Disclosure:**

No significant relationships.

